# Resilience@Work Mindfulness Program: Results From a Cluster Randomized Controlled Trial With First Responders

**DOI:** 10.2196/12894

**Published:** 2019-02-19

**Authors:** Sadhbh Joyce, Fiona Shand, Tara J Lal, Brendan Mott, Richard A Bryant, Samuel B Harvey

**Affiliations:** 1 School of Psychiatry Faculty of Medicine University of New South Wales Randwick Australia; 2 The Black Dog Institute Hospital Road Randwick Australia; 3 Fire and Rescue New South Wales Sydney Australia; 4 University of New South Wales School of Psychology Sydney Australia

**Keywords:** resilience training, workplace mental health, occupational health, well-being, online intervention, employee resilience, health and safety, psychological health, first responders

## Abstract

**Background:**

A growing body of research suggests that resilience training can play a pivotal role in creating mentally healthy workplaces, particularly with regard to protecting the long-term well-being of workers. Emerging research describes positive outcomes from various types of resilience training programs (RTPs) among different occupational groups. One specific group of workers that may benefit from this form of proactive resilience training is first responders. Given the nature of their work, first responders are frequently exposed to stressful circumstances and potentially traumatic events, which may impact their overall resilience and well-being over time.

**Objective:**

This study aimed to examine whether a mindfulness-based RTP (the Resilience@Work [RAW] Mindfulness Program) delivered via the internet can effectively enhance resilience among a group of high-risk workers.

**Methods:**

We conducted a cluster randomized controlled trial (RCT) comprising 24 Primary Fire and Rescue and Hazmat stations within New South Wales. Overall, 12 stations were assigned to the 6-session RAW Mindfulness Program and 12 stations were assigned to the control condition. A total of 143 active full-time firefighters enrolled in the study. Questionnaires were administered at baseline, immediately post training, and at 6-month follow-up. Measurements examined change in both adaptive and bounce-back resilience as well as several secondary outcomes examining resilience resources and acceptance and mindfulness skills.

**Results:**

Mixed-model repeated measures analysis found that the overall test of group-by-time interaction was significant (*P*=.008), with the intervention group increasing in adaptive resilience over time. However, no significant differences were found between the intervention group and the control group in terms of change in bounce-back resilience (*P*=.09). At 6-month follow-up, the group receiving the RAW intervention had an average increase in their resilience score of 1.3, equating to a moderate-to-large effect size compared with the control group of 0.73 (95% CI 0.38-1.06). Per-protocol analysis found that compared with the control group, the greatest improvements in adaptive resilience were observed among those who completed most of the RAW program, that is, 5 to 6 sessions (*P*=.002).

**Conclusions:**

The results of this RCT suggest that mindfulness-based resilience training delivered in an internet format can create improvements in adaptive resilience and related resources among high-risk workers, such as first responders. Despite a number of limitations, the results of this study suggest that the RAW Mindfulness Program is an effective, scalable, and practical means of delivering online resilience training in high-risk workplace settings. To the best of our knowledge, this is the first time a mindfulness-based RTP delivered entirely via the internet has been tested in the workplace.

**Trial Registration:**

Australian New Zealand Clinical Trials Registry ACTRN12615000574549; https://www.anzctr.org.au/Trial/Registration/TrialReview.aspx?id=368296 (Archived by WebCite at http://www.webcitation.org/75w4xtrpw).

## Introduction

### Background

Poor mental health is the leading cause of absenteeism and long-term disability worldwide [1-3], costing the global economy an estimated US $1 trillion each year in lost productivity [4]. Most of the common mental health difficulties experienced by workers, such as depression and anxiety, are both treatable and, in some cases, preventable [5-7]. Policy makers and researchers are now concentrating their efforts on how to address this public health issue, with a specific focus on what can be done within the workplace. Research continues to highlight the important role the workplace can play in the prevention, treatment, and management of common mental health conditions [8]. Developing mentally healthy workplaces requires a multifaceted approach across all levels of an organization and should include proactive and preventative strategies [9].

One proactive approach receiving increased attention from both public and private sectors is individual psychological resilience training. Resilience can be viewed as a dynamic process reflecting a person’s ability to adapt, manage, and recover effectively from stressful experiences and adverse circumstances [10]. Employee resilience has previously been identified as a key component in creating mentally healthy workplaces [9]. An emerging body of research describes positive outcomes from various types of resilience training programs (RTPs) among different groups, including medical specialists, factory workers, nurses, youth workers, and public servants [11-16]. In addition, research among high-risk occupations, such as firefighters, police, paramedics, and military personnel, highlights the benefits of resilience training among those who frequently experience high-stress situations as an inherent aspect of their work [17-19]. Conversely, several larger trials with US army personnel and more recently with London Ambulance Service in the United Kingdom reported more limited improvements following resilience training [20,21].

Determining which RTPs are beneficial to groups such as emergency workers is particularly important for several reasons. First, these workers play a vital role in providing critical services that maintain the health and safety of our communities. Second, given the nature of their work, emergency workers are at higher risk of developing mental health conditions such as depression, anxiety, and alcohol misuse as well as posttraumatic stress disorder (PTSD) [21-24]. Finally, RTPs that are evaluated and found to benefit emergency workers are likely to provide valuable insights in terms of how best to support the mental health of workers in other high-stress occupations (eg, health care, journalism, and military).

### Prior Work

RTPs typically share the common aim of enhancing a person’s ability to manage stressful situations and adverse circumstances more effectively and with greater emotional insight. Although they may share this common goal, RTPs often differ considerably in terms of content, length, and delivery [25]. More recently, researchers have been drawing on evidence-based psychological therapies such as acceptance and commitment therapy (ACT), cognitive behavioral therapy, and mindfulness-based stress reduction to inform program development [11,12,15,26-30]. These RTPs generally include a combination of psychoeducational material, cognitive strategies, and goal setting, with mindfulness training frequently emerging as a core component.

The growing emphasis on mindfulness training within RTPs is understandable, given the large body of research highlighting the positive benefits of this practice on mental health outcomes [31-38]. Several studies have also described the positive impact of mindfulness on psychological resilience [11,12,26,39]. The idea that mindfulness training may serve to enhance psychological resilience is further supported by findings from a recent meta-analysis that found resilience can indeed be enhanced, particularly by programs that involve both cognitive behavioral strategies and mindfulness training [40].

These specific strategies and skills require time to practice and attain proficiency. It is, therefore, unsurprising that most resilience studies completed thus far describe training programs that involve multiple face-to-face training sessions [25,41]. This is particularly challenging for many employers, as taking workers off the job to attend training can result in considerable disruption to business and services. Face-to-face training is also inherently expensive. As well as direct program costs, employers may face many additional expenses, such as travel, accommodation, venue hire, and the cost of replacement staff. Delivering programs in remote areas can also prove to be particularly difficult and expensive. The scalability of RTPs is thus a critical consideration. The more affordable and accessible RTPs can be made, the more likely they are to be implemented on a large scale.

In response to these challenges, workplace mental health researchers have begun to develop and evaluate novel electronic health (eHealth) programs (online, Web-based training) to help improve accessibility and engagement. Results from a recent meta-analysis found that digital mental health interventions in the workplace can improve psychological well-being [38] and work effectiveness among employees [42]. Despite the apparent advantages of eHealth, research examining the efficacy of this approach for resilience training remains sparse. A few trials have examined either a blended approach (ie, programs that combine online and face-to-face resilience training) [12,26] or an online approach with an emphasis on stress reduction and enhancing resilience-related factors [43,44]. As with the main resilience literature to date, these studies vary greatly in their approach to measuring program efficacy, and thus, limited conclusions can be drawn regarding the efficacy of online RTPs.

To address these issues, we developed an interactive e-learning program called the Resilience@Work (RAW) Mindfulness Program. This self-paced intervention aims to enhance psychological resilience among workers. It comprises 6 online training sessions, each taking about 20 to 25 min to complete on a tablet or computer. The program involves mindfulness training, psychoeducation, and a range of skills and strategies drawn from evidence-based therapies, including ACT, mindfulness-based cognitive therapy, and compassion-focused therapy. A more detailed overview of this program was published in a recent pilot study, and it was found that the RAW Mindfulness Program is feasible in a workplace setting and that those using the program showed a trend toward increased resilience and psychological flexibility [45].

### Hypotheses

The main aim of this study was to build upon the initial pilot research and examine whether the RAW Mindfulness Program can improve resilience among a group of high-risk workers, specifically emergency services personnel. Our primary hypothesis is that first responders receiving the RAW Mindfulness Program will have increased resilience following training, compared with the control group. In addition, we will examine the impact of this training on several secondary outcomes, including acceptance and mindfulness skills, as well as resilience resources such as coping, self-compassion, and optimism. To the best of our knowledge, this is the first time an entirely online mindfulness-based RTP has been evaluated via a randomized controlled trial (RCT) with active emergency services personnel.

## Methods

### Cluster Randomization

This study was prospectively registered with the Australian New Zealand Clinical Trial Registry (ANZCTR no: 12615000574549). We conducted a cluster RCT comprising 24 NSW Primary Fire and Rescue and Hazmat Stations. Fire and Rescue New South Wales (FRNSW) is the seventh largest urban fire service in the world and responds to firefighting, rescue, and hazardous material emergencies in Sydney, Australia, and surrounding regional areas. Given the nature of their work, employees are known to have an elevated risk of depression, anxiety, and PTSD [24,46]. Ethics approval was obtained via the Human Research Ethics Committee at the University of New South Wales, Australia. Randomization was at the station level using an online random sequence generator. An external researcher completed the randomization process. Overall, 12 stations were assigned to receive the RAW Mindfulness Program, and 12 stations were assigned to receive the attention-matched control intervention.

### Participants

Potential participants were full-time firefighters working in the 24 Primary Rescue and Hazmat Stations involved in this trial. Participants were informed about the study during a standard well-being talk facilitated by members of the FRNSW Peer Support Team. These talks are performed several times a year as part of FRNSW’s employee well-being initiative. The Peer Support System within NSW Fire and Rescue is a successful and long-running nonhierarchical support service for firefighters. From November 2015 to April 2016, the Peer Support Officers provided a brief presentation on the topic of resilience as well as the aims of the research study and highlighted that participation in the study was voluntary. The presentation slides were provided by the researchers. Following the presentation, firefighters were provided with a participant information sheet and consent form, the study questionnaire, and a stamped address envelop to return their information to the research team if they chose to be a part of this study. Potential participants in the 12 stations randomly assigned to the intervention group were also asked to provide their email address in the consent form to receive information to access the online program. Log-in details were then emailed directly to the participant. When a participant logged into the online RAW program, this was considered as final consent and enrolment in the intervention group. Participants could withdraw at any time from the study. Participants were not aware of the study hypotheses and were blinded to intervention versus control status. Potential participants who were currently engaged in any form of regular psychological therapy with a psychologist and/or psychiatrist were excluded from this study.

### Intervention: Resilience@Work (RAW) Mindfulness Program

Firefighters assigned to the intervention group received the RAW Mindfulness Program. FRNSW granted firefighters who were enrolled in the study permission to access the program at work. Tablets (iPads) were made available in the stations for firefighters to complete the online program. The RAW program is a mindfulness-based intervention, which also draws on ACT and has a significant emphasis on self-compassion and acceptance skills. The intervention comprises 6 online training sessions. Each session takes about 20 to 25 min to complete. A combination of interactive exercises, audio, and animation is used to teach resilience skills. An overview of the core strategies and skills taught in the RAW program is outlined in Table 1. Participants were able to download mindfulness tracks to their own device for continued practice. Each session was provided in a sequential order, with completion of the first module unlocking access to the next and so on. There was a 3-day break in between each session to encourage skills practice. Therefore, the minimum amount of time a participant could complete the training was 3.5 weeks and the maximum was 6 weeks. Participants also had the opportunity to sign up for text message and/or email reminders.

**Table 1:** Overview of skills and topics covered in the Resilience@Work Mindfulness Program.

Session	Resilience topic and skills focus	Mindfulness tracks
1	Introduction to mindfulness, resilience and psychological well-being	Drop anchor; Take 10; Leaves on a stream
2	Mindfulness skills, understanding your reactive mind versus wise mind, recognizing unhelpful mind chatter and managing uncomfortable and unhelpful thoughts (cognitive defusion); Recognizing your values exercise	Mindful Breathing; Defusion Technique; Notice it, Name it, Let it Go (I’m having the thought that…); Defusion technique 2: Thank you Mind
3	Revision of Cognitive Defusion; Introduction to Mindfulness with Emotions, The Reactive Mind and Avoidance, Understanding how values are linked to Emotions; Valued Action check	Creating Space (Mindfulness with emotions); Mindful Body Scan; The Golden Room
4	The problem with Avoidance, Recognizing avoidance strategies versus adaptive strategies	Creating Space; A Mindful Break (mindfulness with words) Surfing Waves
5	Self-care and support, the Compassion myth, barriers to accessing compassion, compassion fatigue, self-compassion actions & resilience, Identifying Mindful Support (compassionate, nonjudgmental and mindful); Valued Action check	A kind and gentle hand (loving-kindness practice); A Safe Place (compassion-focused mindfulness); A bird’s eye view
6	Compassion focused Mindfulness; Gratitude practice, optimism and resilience, identify and celebrate the milestones; creating a personalized action plan to practice skills	Breathing in the Present Moment; A Golden Moment exercise; Being Kind to your old wounds

### Control Condition: Healthy Living Program

Along with the standard FRNSW well-being talk, firefighters who were assigned to the control group received access to the Healthy Living Program (HLP). FRNSW granted permission to firefighters enrolled in the study to access the program while at work. The HLP comprises 6 modules that provide helpful information on a range of health and well-being topics, for example, healthy skin, healthy home environment, and mobile phone use. The self-paced program was available on tablets (iPads) within the station, with each module taking about 20 min to complete.

### Measures

#### Primary Outcome: Measure of Resilience

The primary outcome of this study was resilience. The means by which resilience is best measured remains a topic of considerable discussion in the literature. In their review of resilience scales, Windle et al [47] concluded that there was “no current ‘gold standard.’” They did, however, identify the Connor-Davidson Resilience Scale (CDRISC) and the Brief Resilience Scale (BRS) as 2 of the better measures available to researchers at present. We utilized both measures at baseline and 6-month follow-up in our study to examine 2 inherent constructs of resilience: (1) successful adaptation to stressful life events and circumstances and (2) bounce-back resilience. The 10-item version of the CDRISC was used to examine the ability to successfully adapt and tolerate experiences such as illness, pressure, personal problems, failure, change, and painful feelings [48]. The BRS was employed to specifically examine the concept of *bounce-back* resilience, that is, the ability to recover from stress [49].

##### Connor-Davidson Resilience Scale

Psychological resilience was measured using the validated short-form 10-item version of the Connor-Davidson Resilience Scale (CDRISC_10) [48]. Participants respond to each item on a 5-point scale, ranging from 0 (not true at all) to 4 (true nearly all of the time). The total score ranges from 0 to 40, with a higher score indicative of higher psychological resilience. Previous studies have found CDRISC_10 to be a reliable and valid measure, with Cronbach alpha ranging from .81 to .88 [50,51] and test-retest reliability of .90 at 6 weeks [51,52].

##### Brief Resilience Scale

Bounce-back resilience (the ability to recover from stress) was measured using the 6-item BRS [49]. Items are rated on a 5-point scale ranging from 1 (strongly disagree) to 5 (strongly agree). The total score ranges from 6 to 30, with higher scores signifying greater bounce-back resilience. BRS has demonstrated good internal consistency, with Cronbach alpha ranging from .83 to .90 [49,53].

#### Secondary Outcomes

A number of secondary outcomes were included to examine the processes by which the RAW Mindfulness Program may enhance resilience. Measures of acceptance and mindfulness skills and several resilience resources were administered.

##### Acceptance and Mindfulness Skills

###### Freiburg Mindfulness Inventory

Mindfulness was measured using the short version of the Freiburg Mindfulness Inventory (FMI-14) [54]. Previous studies have found that the FMI-14 is a reliable measure with good internal consistency (Cronbach alpha=.86) [54-56].

###### Cognitive Fusion Questionnaire

The CFQ is a measure of cognitive fusion and defusion, a core component of the ACT model [57]. Higher scores reflect greater thought entanglement. The CFQ has been found to be a reliable and valid measure, with Cronbach alpha ranging from .89 to .93 [57,58].

###### Acceptance and Action Questionnaire

The Acceptance and Action Questionnaire (AAQ-II) is a measure of experiential avoidance and psychological inflexibility. Previous research has found the AAQ-II to be a reliable and valid measure, with a Cronbach alpha of .84 and test-retest reliability of .81 at 3-month follow-up [59].

###### Self-Compassion Scale

The 12-item short form of Self-Compassion Scale (SCS-SF) [60] assesses the level of self-compassion an individual has toward themselves during difficult and challenging times. The measure has high internal consistency (Cronbach alpha=.85) and a nearly perfect correlation with the long form of the SCS [60,61].

##### Resilience Resources

###### Optimism: Life Orientation Test-Revised

The Life Orientation Test-Revised (LOT-R) was used to assess levels of optimism [62]. This 10-item scale examines the extent to which individuals anticipate positive outcomes in the future, with higher scores reflecting greater optimism. The LOT-R has been shown to be a reliable and valid measure (Cronbach alpha=.73) [61,63].

###### Coping: The Brief-Coping Orientation to Problems Experienced

A total of 3 subscales of the Brief-Coping Orientation to Problems Experienced (Brief-COPE) [64] were included *Using Emotional Support* (accepting emotional support, compassion, and sympathy from others), *Using Instrumental Support* (seeking advice, help, or information from others), and *Active Coping* (the process of taking active steps to alter or reduce the impact of a stressor). Higher scores reflect greater use of each coping strategy. These subscales have been found to be reliable measures, with Cronbach alpha values ranging from .64 to .82 [64-66].

###### Sense of Purpose: Life Engagement Test

The extent to which participants value their daily activities and have a sense of life purpose was examined by the 6-item Life Engagement Test (LET) [67]. The LET has previously been found to be a reliable measure, with Cronbach alpha ranging from .72 to .87 [67,68].

### Statistical Analysis

As outlined in the a priori analysis plan recorded in the online trial registry (ANZCTR no: 12615000574549), the primary analysis was undertaken within an intent-to-treat framework utilizing mixed-model repeated measures (MMRM). This approach is recognized as a reliable method of analyzing RCT data [69,70]. MMRM uses all available data and, therefore, does not substitute missing values with estimated values. The dependent variables in these analyses were the absolute values for resilience, as measured by CDRISC_10 and BRS. The relationships between observations at different occasions were modeled using an unstructured variance-covariance matrix. The analysis examined whether the rates of change on resilience scores over time differed significantly between the intervention group and the control group via a group-by-time interaction. In addition, differences between the intervention and control groups at each of the follow-up time points were examined against baseline using planned contrasts. Similar analyses examined the impact of the RAW intervention program compared with the control condition on several secondary outcomes, which included mindfulness/acceptance skills and resilience resources. A priori planned per-protocol analyses were also conducted to assess the effectiveness of the program among participants who completed different numbers of online RAW modules compared with the control group. All tests of treatment effects were conducted using a 2-sided alpha level of .05 and 95% CIs. All analyses were conducted in IBM SPSS version 24. On the basis of our pilot study results of baseline resilience levels [45] and predicted intracluster correlation coefficient of .01, we estimated that a total of 24 fire stations (clusters) including 144 firefighters would need to be recruited to achieve .80 power of detecting a .50 SD improvement in resilience measures with an alpha of .05 (2-sided).

## Results

### Overview

**Figure 1:**
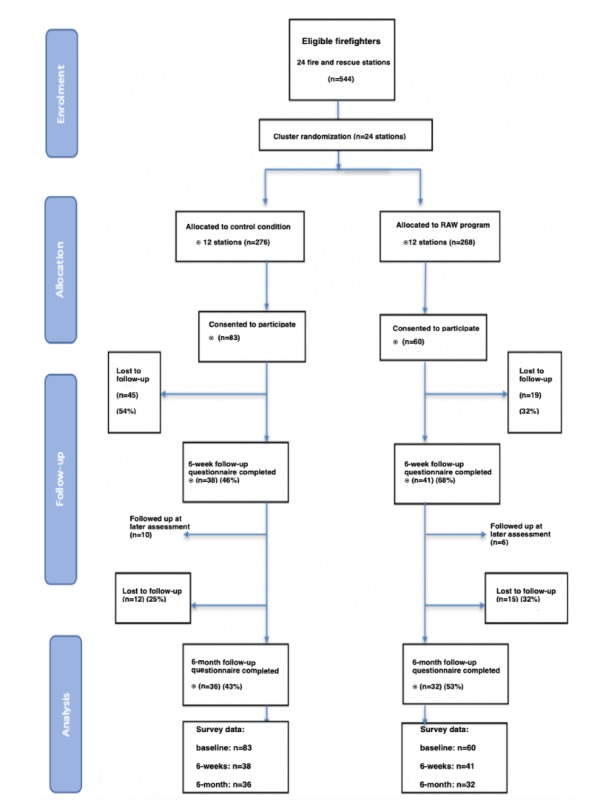
Consolidated Standards of Reporting Trials flow diagram. RAW: Resilience@Work.

This study was conducted in accordance with the Consolidated Standards of Reporting Trials (CONSORT) statement and guideline for transparent reporting of RCTs. Figure 1 outlines the flow of recruitment and loss to follow-up. Overall, a total of 143 firefighters were recruited, with 83 firefighters assigned to the control condition and 60 firefighters to the RAW Mindfulness Program. Of those that were recruited, data for the primary outcome were available for 55.2% (79/143) of the sample at 6-week follow-up. At 6 months, there was a decrease in the follow-up rate to 48.3% (69/143). There was a greater loss to follow-up in the control group at post intervention (54% [45/83] compared with 32% [19/60]; *P*=.01); however, by the 6-month follow-up, this differential loss to follow-up was no longer apparent (43% [36/83] compared with 53% [32/60]; *P*=.24). Loss to follow-up was unrelated to age, gender, years served as a firefighter, baseline measures of resilience, and prior exposure to traumatic events (*P*>.05).

Demographic details of firefighters enrolled in the study are shown in Table 2. In line with recommendations from the CONSORT statement [71], statistical tests of baseline differences were not carried out, as any differences were because of the randomization process and, therefore, by definition, because of random chance. However, observation of the data summarized in Table 2 suggested that the participants in the intervention group were slightly older and more experienced than the participants in the control group and, therefore, may have been exposed to more traumatic incidents across their career. These baseline differences were controlled for in later sensitivity analyses.

**Table 2:** Demographics of enrolled participants at baseline.

Demographics		Intervention group (n=60)	Control group (n=83)
**Gender, n (%)**			
	Male	56 (93)	81 (98)
	Female	4 (7)	2 (2)
Age (years), mean (SD)		43.9 (7.8)	41.1 (9.2)
**Years served as a firefighter, n (%)**			
	1 to 5 years	5 (8)	9 (11)
	6 to 10 years	13 (22)	27 (33)
	11 to 15 years	11 (18)	24 (29)
	16 to 20 years	6 (10)	6 (7)
	More than 20 years	25 (42)	17 (20)
**Number of trauma incidents attended over the course of career, n (%)**			
	1 to 5	3 (5)	14 (17)
	6 to 10	7 (12)	17 (21)
	11 to 15	5 (9)	10 (12)
	16 to 20	6 (10)	5 (6)
	More than 20	37 (64)	37 (45)
**Measures of resilience, mean (SD)**			
	CDRISC_10^a^	28.4 (5.3)	(5.5)
	BRS^b^	22.1 (3.4)	23.0 (3.6)

^a^CDRISC_10: 10-item version of Connor-Davidson Resilience Scale.

^b^BRS: Brief Resilience Scale.

### Resilience@Work Program Engagement

The majority of participants (38/60, 63%) completed more than half the RAW program (mean number of sessions completed was 3.5 out of a possible 6; SD 2.0), equating to 60 to 75 min of training. A total of 22 participants (22/60, 37%) went on to complete 5 to 6 sessions (a total of at least 100 to 120 min of training).

### Resilience

**Figure 2:**
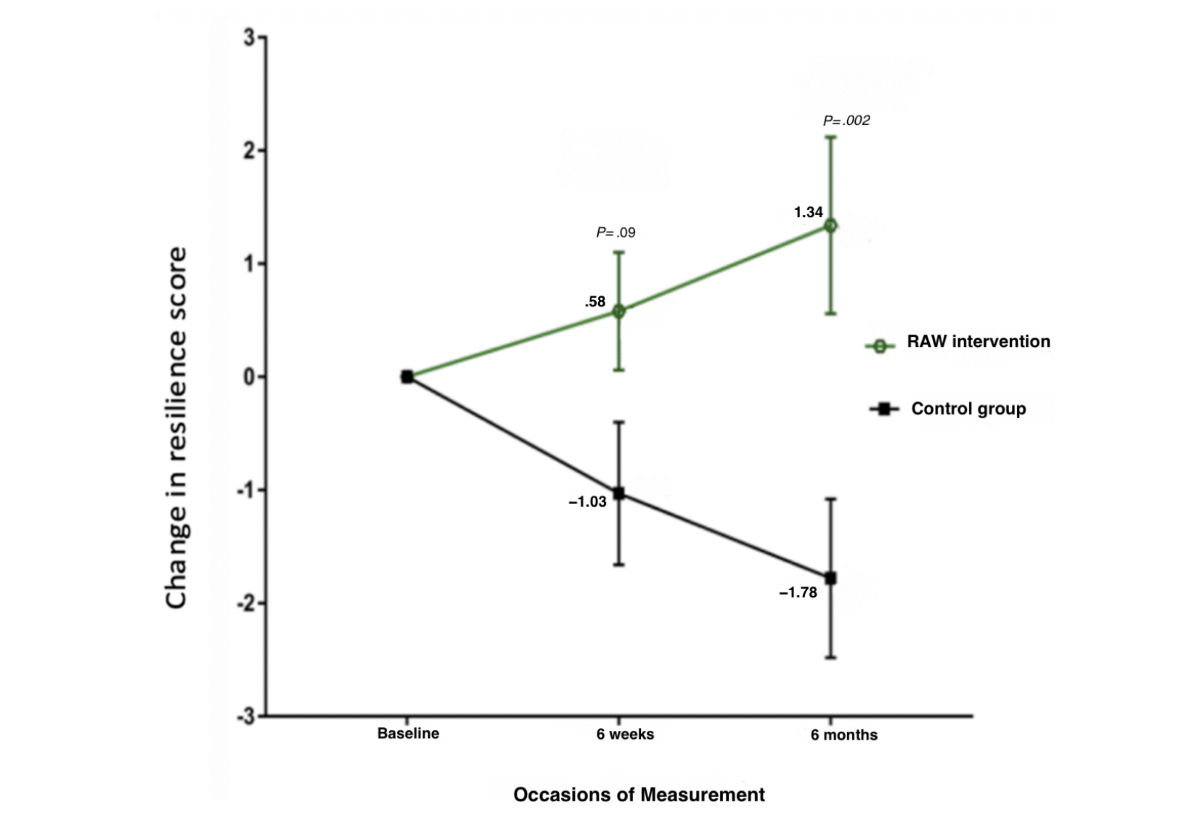
Test of group-by-time interaction (*P*=.01) change on the 10-item version of the Connor-Davidson Resilience Scale. RAW: Resilience@Work.

For the primary outcome, the overall test of group-by-time interaction was significant (*P*=.01), with the intervention group increasing in resilience over time (Figure 2). Although the intervention group increased in resilience at 6 weeks, the difference compared with the control group at this time point fell short of significance (*P*=.09). However, at 6-month follow-up, the intervention group continued to improve in resilience and was significantly different from the control group (*P*=.002). At 6-month follow-up, the group receiving the RAW intervention had an average increase in their resilience score of 1.3, equating to a moderate-to-large effect size compared with the control group of 0.73 (CI: 0.38-1.06). This effect remained when the analysis was repeated with adjustment for baseline age, years of service, and number of traumatic incidents experienced (test of group-by-time interaction, *P*=.008; difference between groups at 6-month follow-up, *P*=.02).

No significant differences were found between the intervention group and the control group in terms of change in bounce-back resilience as measured by BRS (*P*=.09). This finding was replicated when the analysis was repeated to adjust for baseline age, years of service, and number of traumatic incidents experienced (test of group-by-time interaction, *P*=.09).

**Figure 3:**
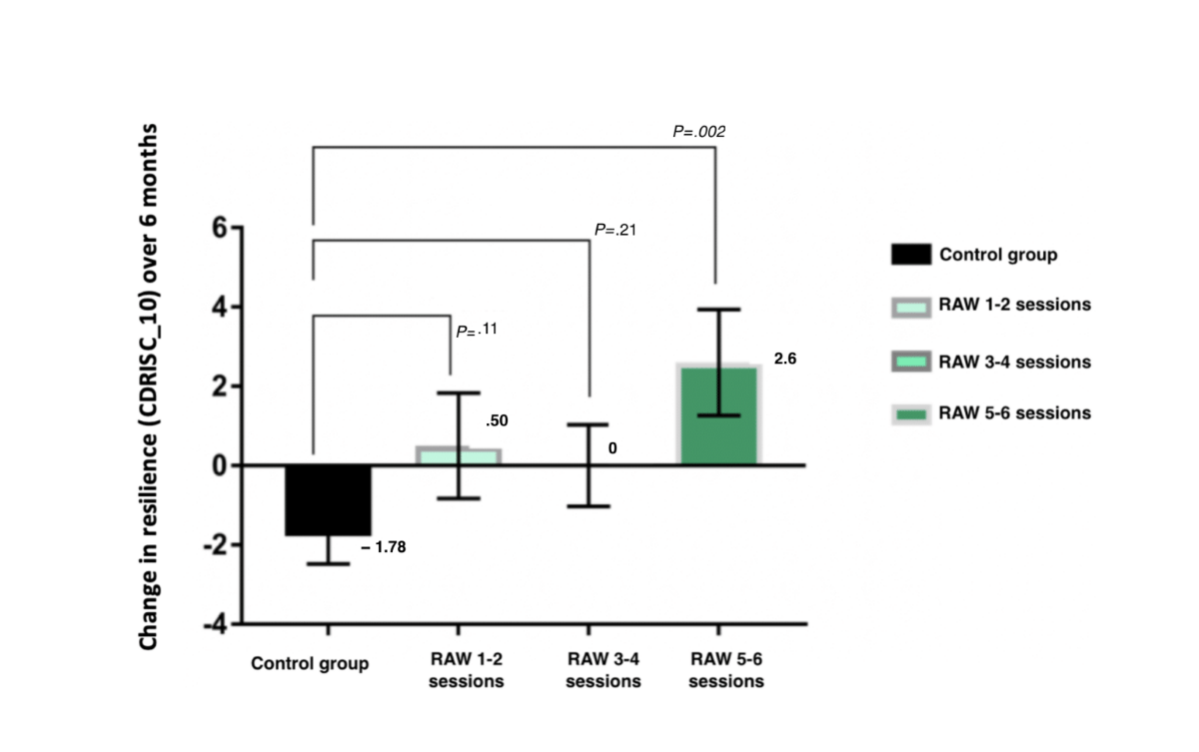
Analysis of primary outcome (change on the 10-item version of the Connor-Davidson Resilience Scale) by number of Resilience@Work sessions. Statistical difference test of significance compared with control group obtained from mixed-methods repeated measures models. RAW: Resilience@Work; CDRISC_10: 10-item version of the Connor-Davidson Resilience Scale.

A per-protocol analysis examined the dose response to the RAW intervention program at 6-month follow-up (Figure 3). Compared with the control group, there was a significant and positive change in CDRISC_10 resilience scores at 6-month follow-up among those who completed most of the RAW program, that is, 5 to 6 sessions (*P*=.002). Within the intervention group, there was no difference between partial and full completers (5-6 sessions) of the RAW program in terms of age (*P*=.20), gender (*P*=.62), and baseline levels of resilience (*P*=.39).

**Table 3:** Mixed-model repeated measures with secondary outcome variables change at 6 weeks and 6 months for intervention and control groups.

Measure		Description	Change at 6 weeks Mean (SE)		Significance test of difference at 6 weeks^a^	Change at 6 months Mean (SE)		Significance test of difference at 6 months^a^
			Control	RAW		Control	RAW	
**Acceptance and mindfulness skills**								
	Cognitive Fusion (CFQ)	Level of thought entanglement	−0.18 (1.07)	−0.85 (0.74)	.25	0.06 (0.89)	−1.65 (1.18)	.40
	Experiential Avoidance(AQQ_II)	Psychological inflexibility/ reactivity, tendency to avoid experiencing internal events	−0.29 (1.38)	−0.83 (0.56)	.31	−0.27 (0.81)	−0.94 (0.81)	.27
	Self-Compassion (SCS-SF)	Level of self-compassion during difficult times	0.89 (1.66)	1.31 (0.95)	.42	0.71 (1.24)	1.47 (1.08)	.99
	Mindfulness (FMI)	Level of mindfulness and present moment awareness	0.38 (1.06)	1.18 (0.87)	.79	0.37 (0.99)	4.16 (1.05)	.09
**Resilience resources**								
	Life Engagement Test (LET)	Sense of purpose in Life	0.00 (0.64)	0.11(0.57)	.83	−0.69 (0.66)	−0.60 (0.53)	.89
	Life Orientation Test- Revised (LOT-R)	Level of optimism	−0.83 (0.55)	1.2 (.43)	.05^a^	−0.86 (0.48)	0.38 (0.68)	.14
	Active Coping (AC)	Personal effort and actions to change and improve current situation	0.11 (0.46)	0.40 (0.43)	.09	−0.56 (0.44)	0.21 (0.43)	.046^a^
	Use of Emotional Support (ES)	Emotional, empathic and comfort from others	−1.35 (0.48)	0.30 (0.36)	.05^a^	−0.40 (0.34)	0.24 (0.30)	.10
	Use of Instrumental Support (IS)	Advice/help from others on what actions to take	−0.47 (0.43)	0.43 (0.36)	.05^a^	−0.29 (0.37)	0.03 (0.38)	.32

^a^Significant at *P*<0.05. Test of difference between groups at each time point were obtained from planned contrasts utilizing unadjusted mixed-model repeated measures analysis.

### Secondary Outcomes

Analyses of change for secondary outcomes are shown in Table 3. Improvements within the intervention group were observed in mindfulness and self-compassion as well as reduced thought entanglement (cognitive fusion) and experiential avoidance; however, these changes were not significantly different from those observed in the control condition. With regard to resilience resources, there were statistically significant improvements noted in optimism (*P*=.05), use of emotional support (*P*=.05), and use of instrumental support (*P*=.05) at 6-week follow-up in the intervention group compared with the control condition; however, this change was not sustained at 6-month follow-up. There was a significant and sustained improvement in active coping among the intervention group when compared with the control group at 6-month follow-up (*P*=.046).

**Figure 4:**
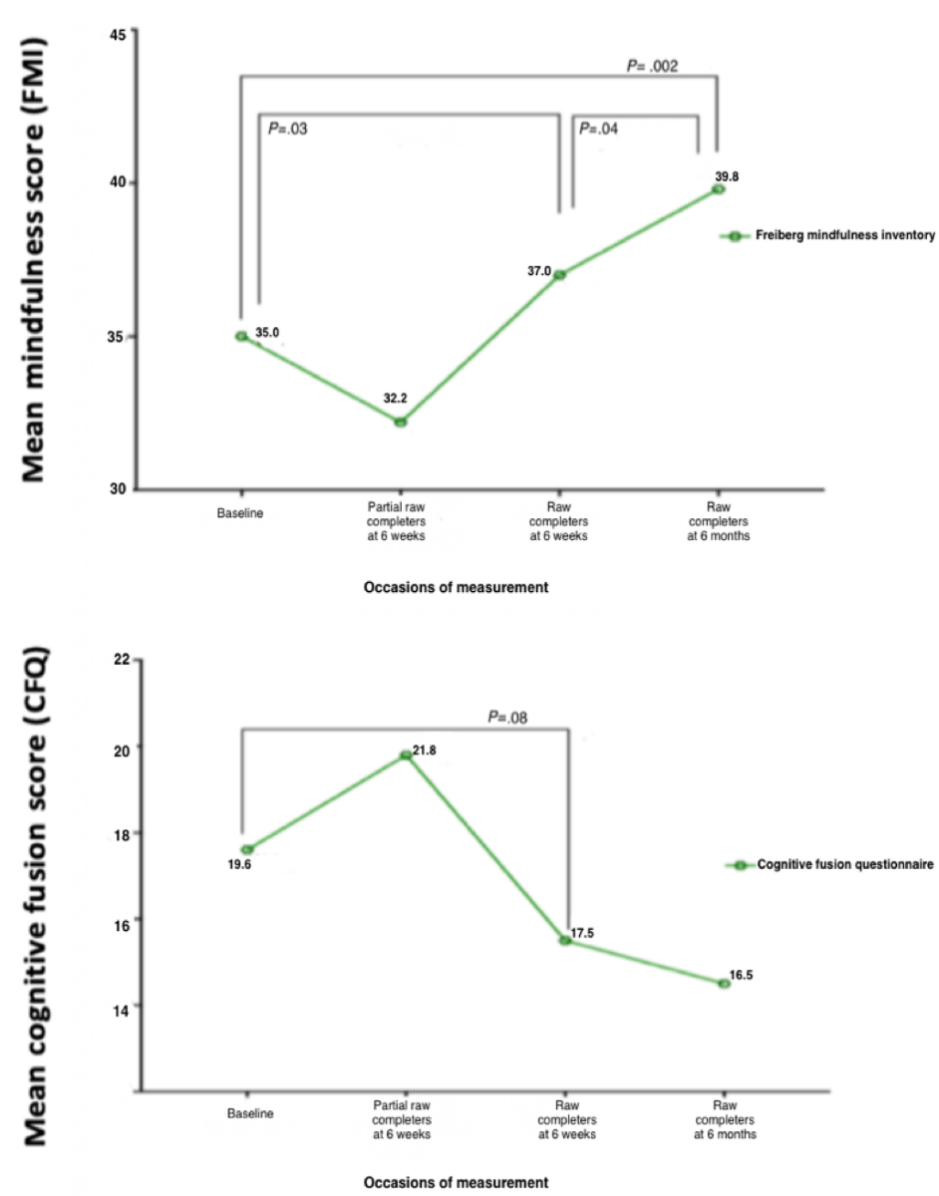
Analysis of change in mindfulness and cognitive fusion scores over time for partial and full program completers. RAW: Resilience@Work; FMI: Freiburg Mindfulness Inventory; CFQ: cognitive fusion questionnaire.

Finally, we examined how mindfulness (FMI) and cognitive defusion (CFQ) skills changed over time for partial completers (1-4 sessions) and completers (5-6 sessions). Change in scores was examined across 3 time points: baseline, 6-week follow up, and 6-month follow-up. The differences between baseline and different follow-up for partial completers and completers were analyzed using paired *t* tests (Figure 4). For RAW program completers, significant improvements were observed in mindfulness skills at 6-week follow-up (*P*=.03) and 6-month follow-up (*P*=.002) compared with baseline. The improvements in mindfulness from 6-week follow-up to 6-month follow-up were also statistically significant (*P*=.046). In addition, there was a trend toward reduced cognitive fusion/thought entanglement (CFQ) at 6-week follow-up for program completers (*P*=.08). However, for partial completers, there was limited change in mindfulness or cognitive defusion.

## Discussion

### Principal Findings

This study is the first ever RCT to test the ability of an entirely online training program to enhance psychological resilience. Our results demonstrate that the RAW Mindfulness Program enhanced adaptive psychological resilience among active firefighters, increasing their ability to adapt successfully in the face of adverse circumstances and situations. Our per-protocol analysis found that the greatest improvements were seen among those who completed all 6 RAW sessions—at least 2 hours of training over a minimum of 3.5 weeks. The results support previous findings that mindfulness-based RTPs can create improvements to individual resilience [40,53,72]. The results also further support previous research regarding workplace eHealth initiatives and the positive impact they can have on employee well-being [38,42]. These findings have important implications for other high-risk groups, with workers such as paramedics, police, defense personnel, doctors, nurses, midwives, and journalists, who may also be likely to benefit from similar interventions.

This study utilized 2 of the most commonly used measures of resilience, along with several measures of resilience-related factors. This approach was in response to the absence of a current gold standard resilience measure [47] and the growing consensus among researchers that resilience is a multifaceted construct [48,73]. The RAW program resulted in significant improvements in resilience as measured by the CDRISC_10; however, no changes were observed on the BRS. It is difficult to establish why RAW led to improvements on the CDRISC_10 and not the BRS. One possible explanation is that our baseline sample was already scoring in the average to high range on the BRS and yet low average to average on the CDRISC. This may have limited the opportunity to enhance bounce-back resilience in this group. This observation supports the notion that these scales are measuring 2 different aspects of resilience and that the RAW program may enhance certain aspects of resilience and not others. Endorsement of the CDRISC_10 reflects an individual’s ability to tolerate experiences such as change, personal problems, illness, pressure, failure, and painful feelings [48,73,74]. In contrast, Smith et al argue that the BRS is currently the only scale that accurately measures resilience in its most basic form as it focuses solely on a person’s ability to *bounce back* or recover from stress rather than the personal qualities that facilitate and promote positive adaptation [49]. Smith et al emphasize that *bounce-back* resilience is particularly important when examining groups that are already unwell or facing health-related stressors, as it reflects the specific ability to recover rather than one’s ability to resist illness. In their review of resilience measures, Windle et al note that although items in the BRS correspond with the ability to recover and cope with challenging circumstances, all 6 items reflect a sense of personal agency [47]. Although personal agency can be viewed as an essential part of resilient adjustment to adversity, it is now well documented that resilience is a dynamic multifaceted construct, with no single factor accurately predicting resilience outcomes [47,74]. For groups of relatively healthy workers who do not identify as being unwell physically or psychologically, focusing on factors that can enhance adaptive resilience may serve a greater purpose than examining factors related to personal agency. The enhancement of adaptive resilience and related resources may be central to such groups’ abilities to manage potentially stressful and challenging situations in the future. This, in turn, may have a direct impact on their overall psychological well-being. Indeed, a recent study of the relationship between baseline resilience and mental health outcomes [75] found that low adaptive resilience among active first responders (as measured by the CDRISC_10) was an accurate predictor of increased PTSD symptoms at 6-month follow-up. Similarly, researchers in the United Kingdom found that low adaptive resilience at baseline (measured by the CDRISC_25) accurately predicted greater symptoms of depression at 2-year follow-up among active paramedics [76].

In terms of the secondary outcomes examined in this study, the RAW program resulted in significant improvements in overall optimism, use of emotional support (seeking empathy and emotional support from others), and use of instrumental support (actively seeking advice and help from others on what actions to take) post training. At 6-month follow-up, there was a significant improvement in the level of active coping (personal effort and actions to change and improve the current situation). This may further explain why improvements were observed on the CDRISC_10 and not the BRS, as the RAW program focused heavily on the acquisition of skills directly aimed at enhancing resilience resources that are more directly measured by the CDRISC. Surprisingly, although an overall trend of improvement in acceptance and mindfulness skills was observed in the RAW group, when compared with the control group, these changes did not reach statistical significance. As this study’s power analysis was based on our primary outcome of resilience, it may have been underpowered to detect a difference on measures of mindfulness and acceptance skills.

Overall, our findings suggest that the RAW program can improve specific resilience resources and aspects of resilience such as distress tolerance, positive adjustment, and perseverance, as measured by the CDRISC_10. It may have less impact on the concept of *bounce back* as measured by the BRS. It is also important to consider why improvements in resilience were more prominent at 6-month follow-up than immediately following training. Time is an essential aspect in any skill’s acquisition, allowing the opportunity for ongoing practice, development, and refinement. Given the greater time to practice and develop their skills, it is consistent that enhanced resilience was seen at 6-month follow-up. These findings are in line with results from a recent meta-analysis and systematic review of RTPs, which found that programs delivered over time can improve resilience, whereas one-off training sessions resulted in little to no improvement [40]. This also speaks to the temporal elements of resilience and the importance of including longer-term follow-up in trials examining the efficacy of RTPs [74]. Resilience studies to date have generally limited data collection time points to posttraining and 3-month follow-up, with minimal good-quality studies including 6-month follow-up data [25,40].

### Limitations

Limitations to this research include that the workforce was a male-dominated high-risk group, thus limiting the generalizability of the findings to lower-risk and gender-balanced occupational groups. It is also important to acknowledge that during the trial period, the self-reported resilience of the control group reduced. It is unknown whether this may be related to their ongoing trauma exposure or whether it may be the natural trajectory of resilience among firefighters over a 6-month period. The latter may be the case, given that participants enrolled in this study were all active, full-time firefighters based at Primary Rescue and Hazmat Stations. These stations are the busiest across the state of New South Wales and frequently respond to serious emergencies, critical incidents, and disaster situations, including motor vehicle accidents, suicide, structural fires, hazard material, and body recovery. In light of these environmental factors and the workplace setting, it is perhaps unsurprising that the overall resilience of firefighters in the control group declined over a 6-month period. Importantly, this highlights the significance of the temporal elements of resilience and the value of measuring it over several time points [74]. A further limitation in the study’s design was the second step in the consent process for the intervention group, that is, logging into the online program. This is a potential source of bias and may have influenced the different rates of loss to follow-up. Another design limitation was the omission of measurements examining personal motivation and skills implementation. Therefore, we have limited insight into how often the skills were used post training and whether they were implemented during certain work situations. It will be critical to include these measurements in future evaluations of the RAW program. It will also be important to examine whether increased resilience can lead to improvements in job-related factors such as productivity, safety, and reduced on-the-job errors.

It is important to also acknowledge the adherence issues in this trial. Although the majority of firefighters (63%) in our study completed over half the program (3.5 out of 6 modules), only 37% of firefighters went on to complete the entire program. Unfortunately, we did not gather specific follow-up information from participants regarding their reasons to stop training. Some potential reasons include poor internet connection within the station, the program being accessible only on tablets or computers, reduced motivation, or limited available time because of competing work responsibilities. It is also possible that a participant may have found certain skills in the early modules helpful and, therefore, felt they did not require additional training. Alternatively, some participants may have found some aspects of the training repetitive and restrictive (each module had to be completed to unlock the next one) and, therefore, lost interest in the program. In future evaluations of the program, it will be important to examine why participants stop engaging in the RAW program and whether having access to the program on other personal devices such as smartphones may increase program adherence. In addition, as noted by recent research [12], providing greater control and flexibility around how participants access the online intervention may well increase program adherence and long-term engagement.

A final limitation is that the primary outcome of resilience was measured by self-report. Although the measures used are the most validated and widely used measures of resilience available, it is important that future studies are able to assess whether changes in self-reported resilience from interventions such as RAW translate into fewer incidents of mental illness over time. It is important to note that the RAW program was specifically aimed at enhancing personal resilience rather than reducing mental health symptoms. Given the emerging literature on the relationship between low resilience and increased risk of future mental health difficulties [75,76], the next key step in this research will be to examine whether improved resilience results in reduced mental health symptomology and whether this reduction is sustained over time.

### Conclusions

Despite these limitations, our study’s findings have important implications. First, mindfulness-based resilience training delivered in an online format can create improvements in adaptive resilience and related resources among high-risk workers, such as firefighters. This is particularly significant, given recent findings that low baseline resilience may be a risk factor for increased mental health symptomology in emergency workers [75,76]. As resilience is a potentially modifiable risk factor for mental health conditions in high-risk groups, programs such as RAW may increase an organization’s ability to play a proactive role in protecting psychological well-being. The RAW Mindfulness Program and similar interventions may also serve to bolster psychological resilience among other high-risk groups, such as military personnel, journalists, nurses, doctors, and midwives. Further large-scale resilience trials would offer the opportunity to measure outcomes across different workplaces and occupations. Research trials incorporating extended follow-ups beyond 6 months would also be beneficial, to examine long-term impacts and the optimum time for retraining to occur.

In conclusion, the RAW Mindfulness Program is an effective, scalable, and practical means of delivering online resilience training to high-risk groups such as first responders. With the benefit of further research and development, this form of online resilience training may serve to enhance mental health on a broad scale, protecting workers who perform some of our society’s most challenging roles.

## Abbreviations

AAQ-II: Acceptance and Action Questionnaire version 2
ACT: acceptance and commitment therapy
Brief-COPE: Brief-Coping Orientation to Problems Experienced
BRS: Brief Resilience Scale
CDRISC_10: 10-item version of the Connor-Davidson Resilience Scale
CFQ: cognitive fusion questionnaire
eHealth: electronic health
FMI: Freiburg Mindfulness Inventory
FRNSW: Fire and Rescue New South Wales
HLP: Healthy Living Program
LET: Life Engagement Test
LOT-R: Life Orientation Test-Revised
MMRM: mixed-methods repeated measures
NSW: New South Wales
PTSD: posttraumatic stress disorder
RAW: Resilience@Work
RCT: randomized controlled trial
RTP: resilience training program
SCS: Self-Compassion Scale

## Appendix

Multimedia Appendix 1 CONSORT-EHEALTH checklist (V 1.6.1).
